# Dihydromyricetin Attenuates TNF-*α*-Induced Endothelial Dysfunction through miR-21-Mediated DDAH1/ADMA/NO Signal Pathway

**DOI:** 10.1155/2018/1047810

**Published:** 2018-02-28

**Authors:** Dafeng Yang, Shenglan Tan, Zhousheng Yang, Pei Jiang, Caie Qin, Qiong Yuan, Ruili Dang, Xiaoxia Yao, Jian Qu, Qiong Lu, Ping Xu, Bikui Zhang, Daxiong Xiang, Lei Chen

**Affiliations:** ^1^Department of Pharmacy, Second Xiangya Hospital, Central South University, Changsha, Hunan 410011, China; ^2^Institute of Clinical Pharmacy, Central South University, Changsha, Hunan 410011, China; ^3^Department of Cardiology, Xiangya Hospital, Central South University, Changsha, Hunan 410008, China; ^4^Department of Pharmacy, The People's Hospital of Guangxi Zhuang Autonomous Region, Nanning, Hunan 530021, China; ^5^Department of Clinical Pharmacy and Pharmacology, Jining First People's Hospital, Jining Medical University, Jining 272000, China; ^6^New Drugs Innovation and Development Institute, Department of Pharmacy, College of Medicine, Wuhan University of Science and Technology, Wuhan, Hubei 430065, China; ^7^Department of Surgery, Second Xiangya Hospital, Central South University, Changsha, Hunan 410011, China

## Abstract

Accumulating studies demonstrate that dihydromyricetin (DMY), a compound extracted from Chinese traditional herb,* Ampelopsis grossedentata*, attenuates atherosclerotic process by improvement of endothelial dysfunction. However, the underlying mechanism remains poorly understood. Thus, the aim of this study is to investigate the potential mechanism behind the attenuating effects of DMY on tumor necrosis factor alpha- (TNF-*α*-) induced endothelial dysfunction. In response to TNF-*α*, microRNA-21 (miR-21) expression was significantly increased in human umbilical vein endothelial cells (HUVECs), in line with impaired endothelial dysfunction as evidenced by decreased tube formation and migration, endothelial nitric oxide synthase (eNOS) (ser1177) phosphorylation, dimethylarginine dimethylaminohydrolases 1 (DDAH1) expression and metabolic activity, and nitric oxide (NO) concentration as well as increased asymmetric dimethylarginine (ADMA) levels. In contrast, DMY or blockade of miR-21 expression ameliorated endothelial dysfunction in HUVECs treated with TNF-*α* through downregulation of miR-21 expression, whereas these effects were abolished by overexpression of miR-21. In addition, using a nonspecific NOS inhibitor, L-NAME, also abrogated the attenuating effects of DMY on endothelial dysfunction. Taken together, these data demonstrated that miR-21-mediated DDAH1/ADMA/NO signal pathway plays an important role in TNF-*α*-induced endothelial dysfunction, and DMY attenuated endothelial dysfunction induced by TNF-*α* in a miR-21-dependent manner.

## 1. Introduction

Vascular endothelium dysfunction in response to proinflammatory cytokines, such as tumor necrosis factor alpha (TNF-*α*), is a crucial pathological alteration contributing to the initiation and development of atherosclerosis (AS) and related complications, one of the leading causes of disability and death worldwide [1]. Endothelial derived nitric oxide (NO), produced by endothelial nitric oxide synthase (eNOS), plays a critical role in maintaining endothelial function and impaired NO biosynthesis is a hallmark of endothelial dysfunction involved in the pathogenesis of AS [2]. Increasing evidence has demonstrated that asymmetric dimethylarginine (ADMA), an endogenous NOS inhibitor, inhibited all three isoforms of NOS, resulting in reduced bioavailability of NO and endothelial dysfunction [3, 4]. Dimethylarginine dimethylaminohydrolases (DDAHs) including DDAH1 and DDAH2 contribute to almost 80% ADMA metabolism and DDAH1 is the crucial enzyme in mediating ADMA in both vascular endothelium and plasma [5, 6]. Evidence from experimental and clinical data demonstrates that decreased DDAH expression and/or activity are associated with endothelial dysfunction and are believed to be the mechanism responsible for ADMA-mediated eNOS impairment [7, 8]. Thus, targeting DDAH/ADMA/NO signaling pathway provides a promising therapeutic strategy for AS.

MicroRNAs (miRNAs), a class of evolutionarily conserved small noncoding nucleotides, have garnered considerable attention not only for their ability to mediate cell function by regulating gene expression at posttranscriptional level, but also for their extracellular presence, such as in circulating blood or urine, raising their potential use as biomarkers for diagnosis and/or prognosis (reviewed in [9–12]). MiR-21, a high expressed miRNA in cardiovascular system, plays an important role in the development of AS by mediating its target genes expression, such as peroxisome proliferators-activated receptor-*α* (PPAR-*α*) and WW domain-containing protein 1 (WWP1), which causes endothelial cells activation and decreases endothelial progenitor cell proliferation [13, 14]. In addition, a significant increased expression of miR-21 was observed in both human AS plaque and plasma [15]. Taken together, these data highlight that miR-21 could be a novel target for pharmacological interventions. Moreover, our previous study demonstrated that DDAH1 was one of the direct targets of miR-21 in endothelial cells [16, 17]. However, whether the deleterious effects of miR-21 on endothelial dysfunction are through mediating DDAH1/ADMA/NO signal pathway, especially under the inflammatory cytokine TNF-*α*, is still not unclear.

Accumulating evidence demonstrates that natural products derived from Chinese traditional herb hold great promise as potential therapeutic drugs for treating cardiovascular diseases [18–20]. For instance, dihydromyricetin (DMY), a compound of dihydroflavonol extracted from the stems and leaves of* Ampelopsis grossedentata*, displays multiple pharmacological effects including antioxidation, anti-inflammation, and antitumor [21–24]. Recently, it was reported that DMY exhibited a protective effect on atherosclerotic lesion formation in both high fat diet-fed ApoE knockout (ApoE^−/−^) and low-density lipoprotein receptor deficient (LDLr^−/−^) mice models [25, 26]. However, the underlying molecule mechanisms involved in the beneficial effects of DMY on atherosclerosis are remaining poorly understood. Recently, Meng et al. [27] found that DMY attenuated angiotensin II-induced rat cardiomyocytes hypertrophy through antioxidative effect in a NO-dependent pathway, indicating that DMY was also involved in NO production. Thus, the aim of present study is to investigate the attenuating effects of DMY on endothelial dysfunction through regulating miR-21 expression and its related DDAH1/ADMA/NO signal pathway.

## 2. Materials and Methods

### 2.1. Human Umbilical Vein Endothelial Cells (HUVECs) Culture and Treatment

HUVECs were isolated from pooled umbilical cords, obtained from newborns in the Second Xiangya Hospital, Central South University (Hunan, China), as described in the website of Center for Excellence in Vascular Biology, Harvard Medical School (the website link: http://vrd.bwh.harvard.edu/core_facilities/harvest_huvec.html). HUVECs were cultured in EGM-2 (Lonza, Switzerland) medium containing 20% fetal bovine serum (Gibco, USA) and grown in humidified atmosphere of 5% CO_2_ in air at 37°C. HUVECs at passages 3–5 were used for experiments. The study was approved by the ethics committee of Second Xiangya Hospital, Central South University.

For TNF-*α*, DMY, and L-NAME (Sigma, USA) treatment, HUVECs (200,000/well or 100,000/well) were plated on 6-well or 12-well plates. After 70–80% confluence, cells were incubated with TNF-*α* (50 ng/ml) for 24 h for protein and RNA and 15 min for phosphorylated eNOS (ser1177). In some experiments, HUVECs were treated with different concentrations of DMY (5, 10, 25, 50, 75, and 100 *μ*M) and nonspecific NOS inhibitor L-nitro arginine methyl ester (L-NAME, 100 *μ*M), all drugs being added 1 h before TNF-*α* administration and throughout the TNF-*α* treatment period. DMY was dissolved in DMSO. For cell transfection, HUVECs (100,000/well) were plated on 12-well plates and transfected with miR-21 agomir, miR-21 antagomir, agomir nonspecific control (NC), and antagomir NC (80 nM, resp.) using Ribo FECT CP (RiboBio, Guangzhou, China) transfection reagent according to the manufacture's instruction when cells reached 70–80% confluence for 24 h. After transfection, the supernatant was replaced to fresh medium containing DMY or L-NAME with or without TNF-*α* for further study.

### 2.2. Measurement of Cell Viability

The cell viability detection was using a CCK-8 kit (Dojindo, Japan) as described elsewhere according to the manufacturer's protocol [28]. Briefly, HUVECs (5,000/well) were plated on a 96-well plate and then incubated with different concentrations of DMY (5, 10, 25, 50, 75, and 100 *μ*M) for 24 h. After treatment, 10 *μ*l CCK-8 solution was added into each well and incubated for another 2 h, the absorbance was determined at 450 nm using an ELISA plate reader (DTX880, Beckman, FL).

### 2.3. Quantitative Real-Time Polymerase Chain Reaction (qRT-PCR)

Total mRNA from cell samples was extracted using Trizol reagent (Takara, Japan) as described elsewhere according to the manufacturer's protocol. cDNA was generated from total RNA using reverse transcription kits (Takara, Japan). Amplification was performed by using ViiA 7 DX real-time PCR system (Applied Biosystem, USA) with SYBR real-time PCR kit (Takara, Japan) and the amplification condition was performed with an initial step at 95°C for 30 s and 40 cycles at 95°C for 5 s, annealing at 60°C for 31 s for each target gene. Results were expressed as the ratios of target genes against glyceraldehyde phosphate dehydrogenase (GAPDH) mRNA for DDAH1 and DDAH2 and the small nuclear RNA U6 for miR-21. The primers for DDAH1, DDHA2, and GAPDH were from Shanghai Sangon Company (Shanghai, China), while the primers for miR-21 and U6 were purchased from RiboBio (Guangzhou, China). The primers used in the amplification were as follows: DDAH1-F, 5′-GCCTGATGACATAGCAGCAA-3′, DDAH-1-R, 5′-CCATCCACCTTTTCCAGTTC-3′; DDAH2-F: 5′-ACAAGGACCCCCGCTAAAA-3′, DDAH2-R, 5′-AAGGGAGTCCCCGTCTTCAA-3′; GAPDH-F, 5′-CTGCACCACCAACTGCTTAG-3′, GAPDH-R, 5′-AGGTCC-ACCACTGACACGTT-3′.

### 2.4. Western Blot

Total protein samples were extracted from each sample after treatment as described in [29] and measured using a Bicinchoninic acid (BCA) kit (Beyontime, Jiangsu, China). Lysates (20 *μ*g each sample) were separated by 10% SDS-PAGE gels and then transferred to PVDF membranes. The membranes were blocked with 5% fat-free milk in TBST for 90 min at room temperature and subsequently incubated with corresponding antibodies as follows: DDAH1 (1 : 1000, Abcam, USA), p-eNOS (ser1177) (1 : 1000, Cell Signaling Technology, USA) and GAPDH (1 : 5000, Sigma, USA) at 4°C overnight. After incubation, membranes were washed three times with TBST and then incubated with HRP-conjugated secondary goat-anti-rabbit IgG antibody for 1 h at room temperature. The signal was detected and ratio of the DDAH1 against GAPDH control was calculated using the Bio-Rad Chemi Doc XRS+ Imaging System (Bio-Rad Biosciences, USA).

### 2.5. Measurement of ADMA Concentration, DDAH Metabolic Activity, and NO Concentrations in Cell Medium and/or Cell Lysates

After treatment, the supernatant was collected and stored at −80°C; cells were washed with cold PBS and then incubated with 200 *μ*l cell lysates buffer at 4°C for 30 min. After incubation, samples were centrifuged at 12,000 rpm for 10 min and the supernatant was collected and stored at −80°C. Cell lysates concentration was determined using a BCA kit. The concentration of ADMA in cell medium and lysates was measured using an ELISA kit (CusaBio, Wuhan, China) according to the manufacture's instruction. The ADMA contents in the cell lysates were normalized to the protein concentration. DDAH metabolic activity was detected using an ELISA kit (CusaBio, Wuhan, China) as described elsewhere according to the manufacture's protocol [30]. The concentration of NO in cell medium was measured using an ELISA kit (Beyotime, Jiangsu, China) according to the manufacture's protocol.

### 2.6. Measurement of Tube Formation and Migration

Endothelial dysfunction was measured by tube formation and migration. HUVEs were firstly treated as mentioned in method Section 2.1. Then cells were used for tube formation and migration assay. For tube formation assay, the wells of a 96-well plate were coated with 60 *μ*l ice-cold Matrigel (BD Bioscience, CA) at 37°C for 1 h. HUVECs (10,000/well) were seeded in EGM-2 with 20% FBS and incubated for 4 h. After incubation, images were analyzed at magnification (20x) using a light microscope (Nikon, Japan). The tube formation ability was qualified by counting the total number of complete tubes. The average in 10 random fields was calculated. A migration assay was performed using transwells (8 *μ*m pore polycarbonate membrane, 24 wells, Costar, USA). HUVECs (100,000/well) were seeded on the upper chamber in FBS free EGM-2 media. Then the transwells were placed into the lower chamber containing 500 *μ*l 10% FBS EGM-2 medium and incubated for 18 h. After incubation, the transwell membranes were fixed with 4% paraformaldehyde at room temperature for 15 min and then stained with 0.1% crystal violet solution (Beyotime, Jiangsu, China). The number of migrated cells was counted manually in 10 random fields from microscopic images of transwell membranes and the average was calculated. The images were taken by using a light microscope (Nikon, Japan).

### 2.7. Statistical Analysis

All data were expressed as mean ± SD. Statistical analysis was performed using Graph Pad Prism (version 6.0) software by unpaired Student's *t* test for two groups and one-way ANOVA for multiple groups. A value of *P* < 0.05 was considered statistically significant.

## 3. Results

### 3.1. DMY Represses TNF-*α*-Induced miR-21 Expression in Primary HUVECs

**Figure 1:**
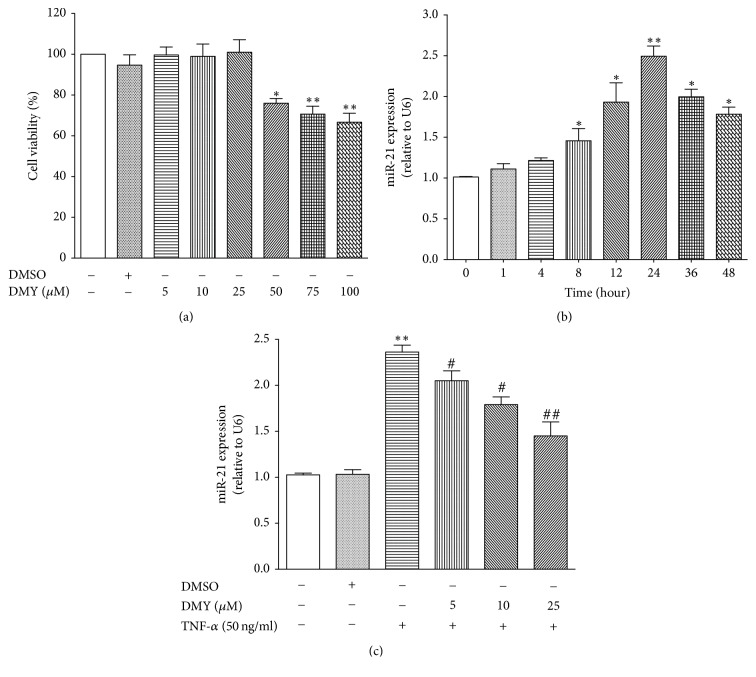
*DMY represses TNF-α-induced miR-21 expression in HUVECs.* (a) The effect of different doses of DMY on HUVECs viability; (b) TNF-*α* induces miR-21 expression in a time-dependent manner; (c) DMY represses TNF-*α*-induced miR-21 expression in a dose-dependent manner. Data was expressed as mean ± SD, *n* = 3–6, ^*∗*^*P* < 0.05 versus control, ^*∗∗*^*P* < 0.01 versus control, ^#^*P* < 0.05 versus TNF-*α*, and ^##^*P* < 0.01 versus TNF-*α*.

To investigate the protective effect of DMY on TNF-*α*-induced endothelial dysfunction, we first evaluated whether DMY had cytotoxic effect on HUVECs using CCK-8 assay to assess the cell viability. As shown in Figure 1(a), DMY at different concentrations (5, 10, and 25 *μ*M, resp.) had no influence on cell viability, while cell viability was decreased when treated with high doses of MDY (50, 75, and 100 *μ*M, resp.). Thus, we selected low doses of DMY (5, 10, and 25 *μ*M) in the subsequent experiments. We next found that, in response to TNF-*α* stimulation, miR-21 expression was remarkably increased after 8 h and had a robust expression at 24 h (almost 2.5-fold) (*P* < 0.05 for 8 h, 12 h, 36 h, and 48 h, resp.; *P* < 0.01 for 24 h) (Figure 1(b)). In contrast, treatment with DMY (5, 10, and 25 *μ*M) significantly repressed TNF-*α*-induced miR-21 expression at 24 h, and DMY at dose of 25 *μ*M had the most profound effect (Figure 1(c)). Collectively, our data demonstrated that miR-21 expression was increased in response to stimulation of inflammatory cytokine TNF-*α* after 24 h, whereas DMY in a dose-dependent manner blockade of TNF-*α*-induced miR-21 expression in primary HUVECs.

### 3.2. DMY Attenuates TNF-*α*-Induced Primary HUVECs Dysfunction through Repressing miR-21 Expression

**Figure 2:**
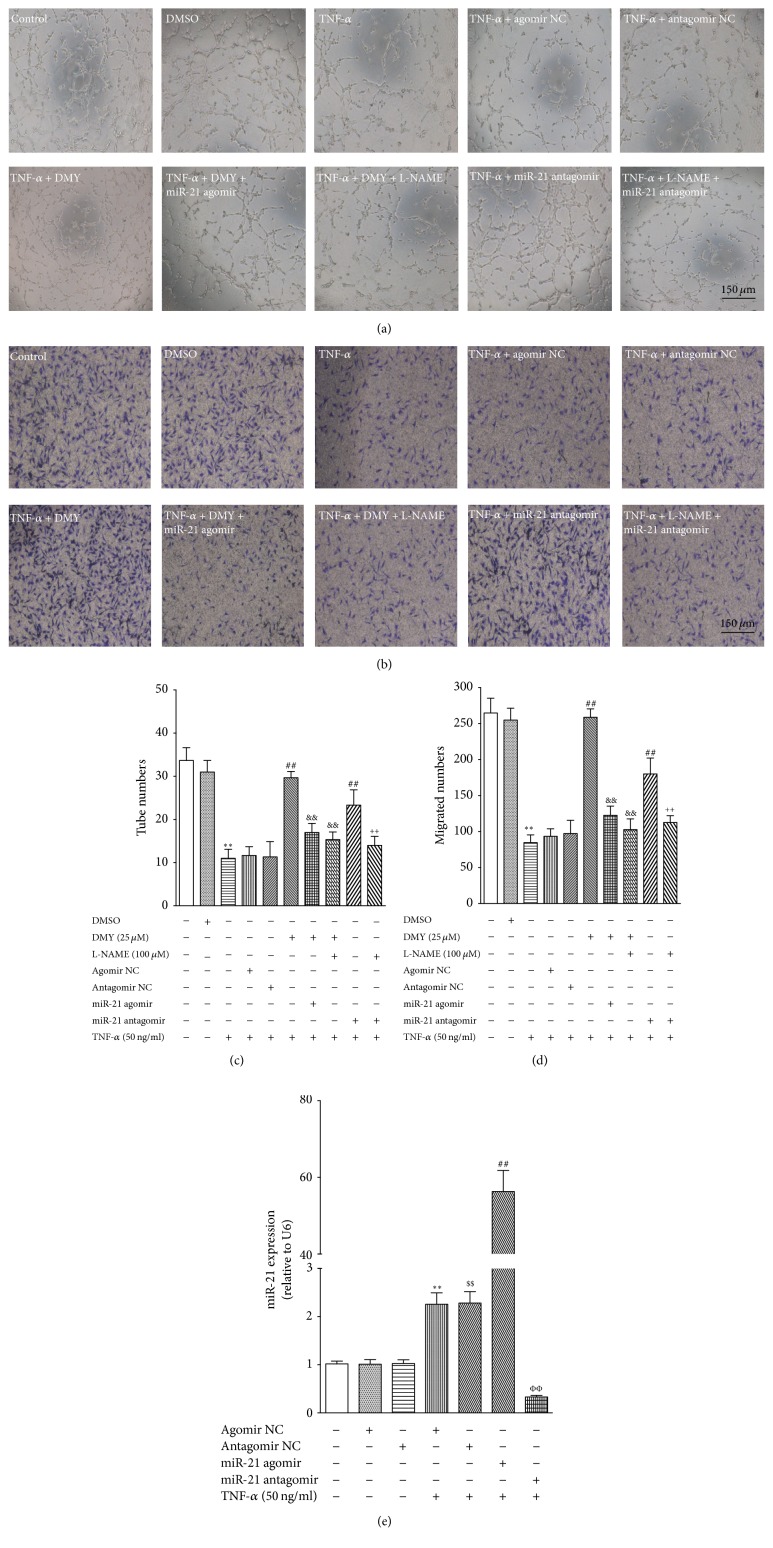
*DMY attenuates TNF-α-impaired tube formation and migration in a miR-21 and NO-dependent manner in HUVECs*. (a) Representative images show the effect of DMY, miR-21 agomir and antagomir, and L-NAME on tube formation induced by TNF-*α* (magnification 20x; scale bar, 150 *μ*m); (b) representative images show the effect of DMY, miR-21 agomir and antagomir, and L-NAME on migrated cells on transwell membranes induced by TNF-*α* (magnification 20x; scale bar, 150 *μ*m). (c) The number of tubes was counted in (a). (d) The number of migrated cells was counted in (b). (e) The transfected efficiency of miR-21 agomir and antagomir on miR-21 expression in HUVECs treated with TNF-*α*. Data was expressed as mean ± SD, *n* = 3, ^*∗∗*^*P* < 0.01 versus control, ^##^*P* < 0.01 versus TNF-*α*, ^&&^*P* < 0.01 versus TNF-*α* + DMY (25 *μ*M), and ^++^*P* < 0.01 versus TNF-*α* + miR-21 antagomir. ^*∗∗*^*P* < 0.01 versus agomir NC, ^$$^*P* < 0.01 versus antagomir NC, ^##^*P* < 0.01 versus TNF-*α* + miR-21 agomir NC, and ^ΦΦ^*P* < 0.01 versus TNF-*α* + miR-21 antagomir NC in (e).

It is well known that impaired tube formation and migration are key hallmarks of endothelial dysfunction. Compared with control or vehicle-treated control, TNF-*α* treatment resulted in a significant endothelial dysfunction as evidenced by decreased tube formation and migration; however, these insults were attenuated by treatment with DMY (25 *μ*M, *P* < 0.01) (Figures 2(a), 2(b), 2(c), and 2(d)). Given that miR-21 expression was elevated in response to TNF-*α* stimuli (Figure 1(b)) and contributed to causing endothelial function, while DMY could repress its expression, we next investigated whether the protective effect of DMY on endothelial function was dependent on the inhibition of miR-21 expression. To answer this question, mir-21 agomir and antagomir were used to gain- and loss-of-function in the subsequently studies. As shown in Figure 2, the ability of tube formation and migration was improved in HUVECs transfected with miR-21 antagomir than in cells transfected with the antagomir negative control. Meanwhile, overexpression of miR-21 using agomir abolished DMY attenuated endothelial dysfunction in TNF-*α* treated HUVECs (Figures 2(a), 2(b), 2(c), and 2(d)). The expression of miR-21 in agomir or antagomir transfected HUVEC cells was confirmed by qPCR (Figure 2(e)). Taken together, these findings suggest that elevated miR-21 expression contributes to TNF-*α*-induced endothelial dysfunction and the attenuating effects of DMY on TNF-*α*-induced HUVECs dysfunction depend on the repressing miR-21 expression.

### 3.3. DMY Increases NO Generation by Decreasing Both Intracellular and Extracellular ADMA Concentration in TNF-*α* Treated HUVECs

**Figure 3:**
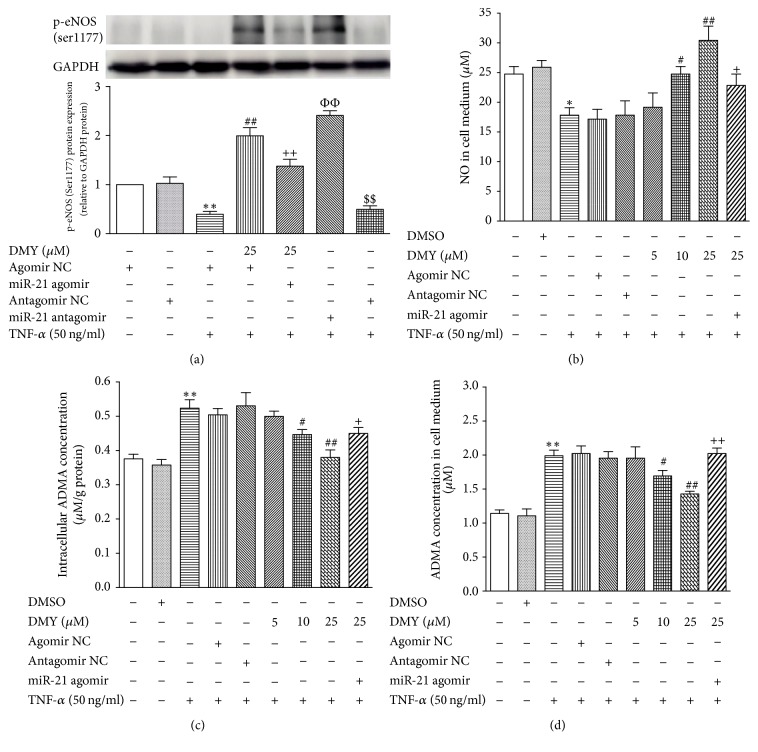
*DMY increases NO generation and decreases intracellular and extracellular ADMA concentration in a miR-21-dependent manner in TNF-α treated HUVECs.* (a) DMY increases eNOS (ser1177) phosphorylation in a miR-21-dependent manner; (b) DMY increases NO concentration in cell medium induced by TNF-*α* in a miR-21-dependent manner; (c) DMY decreases intracellular ADMA concentration induced by TNF-*α* in a miR-21-dependent manner; (d) DMY decreases extracellular ADMA concentration induced by TNF-*α* in a miR-21-dependent manner. Data was expressed as mean ± SD, *n* = 3, ^*∗*^*P* < 0.05 versus control, ^*∗∗*^*P* < 0.05 versus control, ^#^*P* < 0.05 versus TNF-*α*, ^##^*P* < 0.05 versus control, ^+^*P* < 0.05 versus TNF-*α* + DMY (25 *μ*M), and ^++^*P* < 0.01 versus TNF-*α* + DMY (25 *μ*M). ^*∗∗*^*P* < 0.01 versus agomir NC, ^$$^*P* < 0.01 versus antagomir NC, ^##^*P* < 0.01 versus TNF-*α* + miR-21 agomir NC, and ^ΦΦ^*P* < 0.01 versus TNF-*α* + miR-21 antagomir NC in (a).

Given that NO plays an important role in maintaining endothelial function and its production is largely mediated by ADMA, we next examined the NO and ADMA concentration after TNF-*α* stimulation with or without DMY treatment. Compared with the control, TNF-*α* significantly decreased eNOS (ser1177) phosphorylation and NO production and increased both intracellular and extracellular ADMA concentration (Figure 3). In contrast, in response to TNF-*α*, treatment with DMY enhanced eNOS (ser1177) phosphorylation and NO production and decreased both intracellular and extracellular ADMA concentration (*P* < 0.05 for 10 *μ*M; *P* < 0.01 for 25 *μ*M) (Figure 3). However, the effects of DMY on eNOS (ser1177) phosphorylation, ADMA, and NO metabolism were abrogated by overexpression miR-21 (Figure 3). In addition, L-NAME, a nonspecific NOS inhibitor, also abolished DMY and mir-21 antagomir-mediated protective effect by decreasing NO generation (Figures 2(a)–2(d)). These data suggested that DMY improves TNF-*α*-induced HUVECs dysfunction through decreasing ADMA concentration and increasing NO production, and the ameliorated effects are through an NO-dependent manner.

### 3.4. DMY Upregulated DDAH1 Expression and Enhanced DDAH Metabolizing Activity through Repressing miR-21 Expression in TNF-*α* Treated HUVECs

**Figure 4:**
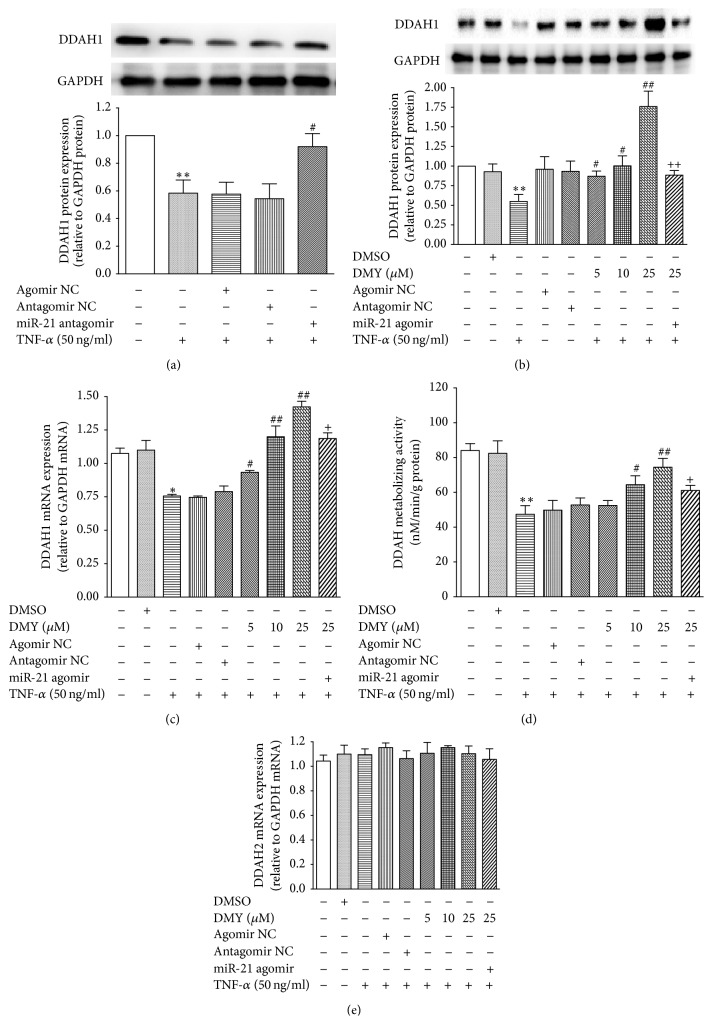
*DMY increases DDAH1 expression and enhances DDAH metabolizing activity in a miR-21-dependent manner in TNF-α treated HUVECs*. (a) Blockade of miR-21 expression increases DDAH1 protein expression induced by TNF-*α*; (b) DMY increases DDAH1 protein expression induced by TNF-*α* in a miR-21-dependent manner; (c) DMY increases DDAH1 mRNA expression induced by TNF-*α* in a miR-21-dependent manner; (d) DMY increases DDAH metabolic activity induced by TNF-*α* in a miR-21-dependent manner; (e) both TNF-*α* and DMY have no effect on DDAH2 mRNA expression. Data was expressed as mean ± SD, *n* = 3, ^*∗*^*P* < 0.05 versus control, ^*∗∗*^*P* < 0.01 versus control, ^#^*P* < 0.05 versus TNF-*α*, ^##^*P* < 0.05 versus TNF-*α*, ^+^*P* < 0.05 versus TNF-*α* + DMY (25 *μ*M), and ^++^*P* < 0.05 versus TNF-*α* + DMY (25 *μ*M).

DDAHs including DDAH1 and DDAH2 play an important role in mediating both intracellular and extracellular ADMA concentration by inactivating ADMA metabolism, resulting in increased NO production. Our previous study identified that DDAH1 was a specific and direct target of miR-21 in HUVECs [16, 17]. In the current study, we find that DMY represses TNF-*α*-induced miR-21 expression and increases NO production (Figures 1 and 2). Thus, we hypothesize that DMY attenuates TNF-*α*-induced endothelial dysfunction through mediating miR-21/DDAH1/ADMA cascade, which in turn results in enhancing NO production. As shown in Figure 4(a), TNF-*α* decreased DDAH1 protein expression, whereas inhibition of miR-21 expression using miR-21 antagomir rescued DDAH1 expression, suggesting that DDAH1 was also a direct target under TNF-*α* treatment. We next investigate whether the effects of DMY on DDAH1 expression and function resulted from repressing miR-21 expression. As shown in Figures 4(b) and 4(c), DMY significantly upregulated DDAH1 expression in both protein (*P* < 0.05 for 5, 10 *μ*M; *P* < 0.01 for 25 *μ*M) and mRNA level (*P* < 0.05 for 25 *μ*M) under TNF-*α* stimulation. Meanwhile, DDAH metabolizing activity assay showed that DDAH activity was also enhanced in HUVECs cotreated with DMY (*P* < 0.05 for 10 *μ*M; *P* < 0.01 for 25 *μ*M) than in cells in presence of TNF-*α* (Figure 4(d)). However, these effects were abolished when overexpressing miR-21 using agomir (Figures 4(b), 4(c), and 4(d)). We also found that both TNF-*α* and DMY did not affect DDAH2 expression (Figure 4(e)). Collectively, these results demonstrate that DMY attenuates TNF-*α*-induced endothelial dysfunction through mediating miR-21/DDAH1/ADMA cascade.

## 4. Discussion

Increasing data from experimental and clinical studies demonstrate that Chinese traditional herb hold great promise as potential therapeutic drugs for treating cardiovascular diseases, including AS. In the present study, we provided evidence that DMY attenuated TNF-*α*-induced endothelial dysfunction through downregulation of miR-21 expression, resulting in an increased expression and activity of DDAH1, which in turn leads to decreasing both intracellular and extracellular ADMA levels and enhancing eNOS (ser1177) phosphorylation and NO production. In supporting, overexpression of miR-21 using miR-21 agomir or inhibition NO production using L-NAME abolished the protective effects of DMY on TNF*α*-induced HUVECs dysfunction. In light of these findings, these data suggested, for the first time, that miR-21-mediated DDAH1/ADMA/NO signal pathway plays an important role in endothelial dysfunction induced by TNF-*α*, and one of the potential mechanisms behind the attenuating effects of DMY may be through regulating miR-21 expression.

Accumulating studies show that miRNAs expression is often tissue- and/or cell-type-specific and hence targets and functional specificity in different cell types or tissues [31–33]. MiR-21 is highly expressed in cardiovascular system and an upregulated miR-21 expression strongly correlates with progression of AS. Clinical studies demonstrated that miR-21 was higher in atherosclerotic plaque compared to nonatherosclerotic artery [15] as well as in plasma of patients with AS [34, 35]. In high fat diet feed ApoE^−/−^ mice model, Notoginsenoside R1 (NR1), a naturally compound from Chinese herb, attenuated atherosclerotic lesion formation by reducing lipid deposition, fibrosis, and oxidative stress, which occurred concomitantly with the downregulation of miR-21 [36]. In contrast, elevated miR-21 expression induced by oscillatory shear stress or ox-LDL resulted in endothelial cell activation, including enhanced expression of vascular cell adhesion molecule-1 (VCAM-1), monocyte chemotactic protein-1 (MCP-1), and increased TNF-*α* release by targeting peroxisome proliferators-activated receptor in endothelial cell activation tensin homolog (PTEN) [13, 37]. In the current study, we found that miR-21 expression was significantly elevated in response to TNF-*α* in HUVECs, as well as impaired endothelial function, including reduced NO concentration and decreased cell migration and tubule formation. However, these were remarkably improved by using miR-21 antagomir, a chemical inhibitor targeting specific miRNA, resulting in decreased miR-21 expression. As expected, treatment with DMY attenuated TNF-*α*-induced HUVECs dysfunction, in line with repressed miR-21 expression, whereas rescued miR-21 expression using agomir abrogated the protective effects of DMY on HUVECs function in response to TNF-*α*. Taken together, these findings demonstrated that miR-21 contributed to TNF-*α*-induced HUVECs dysfunction and the attenuating effects of DMY on endothelial dysfunction induced by TNF-*α* dependent on repressing miR-21 expression.

Impaired NO homeostasis caused by ADMA led to endothelial cells dysfunction, which contributes to the initial process of AS [2, 3]. Evidence from clinic and laboratory studies demonstrated that concentration of ADMA acted as a marker and/or producer of endothelial dysfunction [38–42]. Therefore, ADMA becomes an attractive target in the pathogenesis of AS. Recently, Meng et al. [27] found that DMY attenuated angiotensin II-induced cardiomyocytes hypertrophy by increasing the bioavailability of NO through eNOS phosphorylation. This DMY-mediated effect on hypertrophy could be blocked using a nonspecific NOS inhibitor, which suggested that DMY affects the NO production system. However, the mechanism how DMY increases NO production is still not fully understood. In the present study, we found that, in response to TNF-*α* stimuli, the eNOS phosphorylation and NO concentration were expected to decrease, while both intracellular and extracellular ADMA concentration increased. In contrast, in TNF-*α*-stimulated HUVECs, treatment with DMY remarkable increased eNOS phosphorylation and NO production at 25 *μ*M, whereas it decreased both intracellular and extracellular ADMA concentration. However, these protective responses were abolished by administered miR-21 agomir, resulting in an increased ADMA expression. In addition, NOS inhibitor, L-NAME, also abolished DMY's protective effects on TNF*α*-induced suppression of cell migration and tubule formation, suggesting that DMY attenuated TNF-*α*-induced endothelial dysfunction in a NO-dependent manner. Collectively, these data suggest that DMY attenuates TNF-*α*-induced endothelial dysfunction maybe through downregulation of miR-21 expression and reduction of ADMA levels, resulting in enhanced eNOS phosphorylation and NO production.

The result of the present study demonstrated that DMY decreased both intracellular and extracellular ADMA concentration induced by TNF-*α* in a miR-21-dependant manner, resulting in attenuated endothelial dysfunction. However, the link between miR-21 and ADMA under TNF-*α* and DMY condition is still unknown. It is well known that miRNAs function by directly binding to 3′UTR of direct targeted mRNA sequences, leading to reduction of protein expression and in turn are an important regulator in mediating cell function. DDAHs including DDAH1 and DDAH2 are key enzymes involved in the inactivation of ADMA in the body, and DDAH1 is proved to be the critical enzyme responsible for ADMA metabolism [43, 44]. In our previous study, we identified that DDAH1 was a direct target gene of miR-21 under 4-hydroxynonenal (4-HNE) and without stimulation in HUVECs [16, 17]. Our current study found that TNF-*α* inhibited DDAH1 expression at both protein and mRNA level, which is in line with an increased miR-21 expression. In contrast, knockdown miR-21 expression rescued the DDAH1 expression in TNF-*α*-treated primary HUVECs. These suggested that DDAH1 expression is also regulated by miR-21 under TNF-*α* stimuli. We next found that DMY increased the expression of DDAH1 mRNA and protein, as well as the DDAH metabolic activity, in HUVECs treated with TNF-*α*. However, these effects were abolished when overexpression of miR-21 is using agomir. Taken together, these findings indicated that DMY repressed miR-21 expression, which subsequently increased DDAH1 expression and in turn decreased ADMA concentration and increased NO production.

## 5. Conclusions

**Figure 5:**
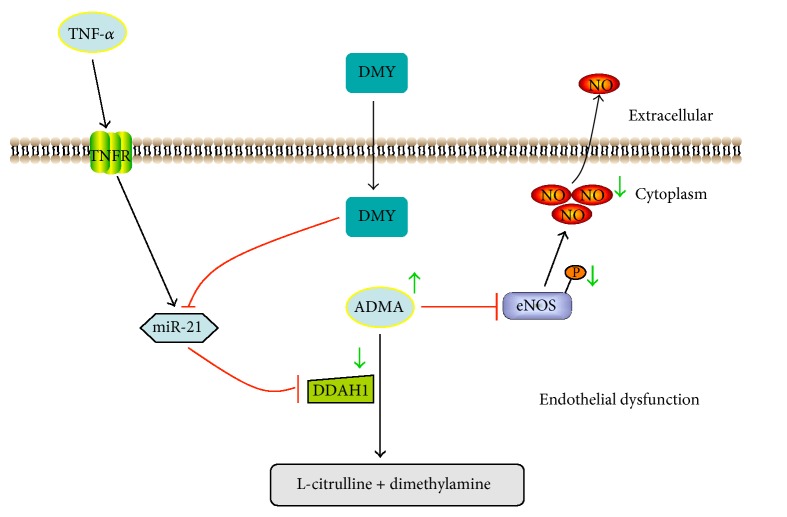
*Schema of mechanism of DMY attenuates TNF-α-induced endothelial dysfunction*. In response to TNF-*α*, miR-21 expression is increased and inhibits DDAH1, an effect that increases downstream ADMA concentration, which resulted in decreasing phosphorylation of eNOS and NO production and led to endothelial dysfunction. In contrast, DMY represses TNF-*α*-induced miR-21 expression and restores endothelial function.

In conclusion, the present study in HUVECs demonstrated that miR-21-mediated DDAH1/ADMA/NO signal pathway plays an important role in TNF-*α*-induced endothelial dysfunction, and DMY attenuated endothelial dysfunction induced by TNF-*α* in a miR-21-dependent manner (Figure 5). Taken together, these findings illuminated the potential mechanism how DMY prevented endothelial dysfunction and suggested that DMY might be a complementary therapeutic drug for the treatment with AS.
